# Live Cell Detection of Poly(ADP-Ribose) for Use in Genetic and Genotoxic Compound Screens

**DOI:** 10.3390/cancers14153676

**Published:** 2022-07-28

**Authors:** Christopher A. Koczor, Aaron J. Haider, Kate M. Saville, Jianfeng Li, Joel F. Andrews, Alison V. Beiser, Robert W. Sobol

**Affiliations:** 1 Department of Pharmacology, College of Medicine, University of South Alabama, Mobile, AL 36688, USA; cakoczor@southalabama.edu (C.A.K.); mkm1325@jagmail.southalabama.edu (K.M.S.); jianfengli@southalabama.edu (J.L.); avbeiser@southalabama.edu (A.V.B.); 2 Mitchell Cancer Institute, University of South Alabama, Mobile, AL 36604, USA; aaronhai@uab.edu (A.J.H.); jandrews@southalabama.edu (J.F.A.)

**Keywords:** Poly(ADP-ribose), PAR, LivePAR, WWE, split luciferase, BER, BRCA2, DNA damage

## Abstract

**Simple Summary:**

Poly(ADP-ribose) (PAR) functions in genome repair, cell metabolism, and cancer treatment; however, in vivo molecular tools to assess PAR levels are not readily available. Here, we describe two methods to measure PAR with high sensitivity in real time in vivo using the PAR binding domain (termed WWE) of Ring Finger Protein 146 (RNF146). In response to genotoxic stress, the WWE-EGFP fusion protein, termed LivePAR, displays nuclear enrichment enabling quantitative and visual detection of PAR formation. Additionally, a split luciferase assay using the same WWE domain facilitates rapid PAR quantitation in a plate format and provides a tool for chemotherapeutic candidate compound screening. Both tools can be used to identify genetic determinants of PAR formation and help in identifying compounds that alter PAR formation or degradation.

**Abstract:**

Poly(ADP-ribose) (PAR) is a molecular scaffold that aids in the formation of DNA repair protein complexes. Tools to sensitively quantify PAR in live cells have been lacking. We recently described the LivePAR probe (EGFP fused to the RNF146-encoded WWE PAR binding domain) to measure PAR formation at sites of laser micro-irradiation in live cells. Here, we present two methods that expand on the use of LivePAR and its WWE domain. First, LivePAR enriches in the nucleus of cells following genotoxic challenge. Image quantitation can identify single-cell PAR formation following genotoxic stress at concentrations lower than PAR ELISA or PAR immunoblot, with greater sensitivity to genotoxic stress than CometChip. In a second approach, we used the RNF146-encoded WWE domain to develop a split luciferase probe for analysis in a 96-well plate assay. We then applied these PAR analysis tools to demonstrate their broad applicability. First, we show that both approaches can identify genetic modifications that alter PARylation levels, such as hyper-PARylation in BRCA2-deficient cancer cells. Second, we demonstrate the utility of the WWE split luciferase assay to characterize the cellular response of genotoxins, PARP inhibitors, and PARG inhibitors, thereby providing a screening method to identify PAR modulating compounds.

## 1. Introduction

Preservation of genomic integrity is key to phenotype maintenance and cell survival, while loss of genomic integrity is a significant step in oncogenesis [1]. There are multiple DNA damage repair pathways that facilitate reparation of DNA lesions, including the base excision repair (BER) pathway, responsible for the repair of base lesions such as those caused by oxidative and alkylative damaging compounds [2,3,4]. The recruitment and assembly of BER proteins at the site of DNA damage is facilitated by poly(ADP-ribose) polymerase 1 (PARP1) and PARP2 via the formation of poly(ADP-ribose) (PAR) chains [5,6,7,8]. PAR serves as the scaffold to help recruit multiple repair-associated proteins via their encoded PAR binding domains (PBDs). At least 10 classes of PBDs have been identified to date that display different binding stringency as well as different binding moiety recognition within the PAR chain [9]. Upon completion of DNA repair, PAR chains are degraded by poly(ADP-ribose) glycohydrolase (PARG) and ADP-ribosylhydrolase 3 (ARH3), promoting disassembly of the associated repair complexes [10,11]. 

In addition to its role in BER, PARP and PAR are important targets in recent and emerging cancer therapies due to the demonstrated synthetic lethality of PARP inhibition in cells with BRCA1/BRCA2 mutations or deletions [12,13]. While the exact mechanism is still under investigation, recent reports have suggested PARP1, and BRCA1/2 regulate the generation of single strand breaks formed during Okazaki fragment processing [14,15,16,17]. It has also been shown that loss of BRCA2 is associated with hyperactivation of PARP1 (with no associated change in PARP1 protein levels) and increased PAR synthesis, supporting increasing reliance on PAR in BRCA-deficient cells with compromised homologous recombination [18], though the role of HR deficiency in chemotherapeutic sensitivity has been challenged [19]. Additionally, while PARP inhibitors (PARPi) have been used to treat BRCA-mutant tumors with success [20], reversion/restoration of BRCA1/2 expression can occur, enabling tumors to evade PARP inhibitor (PARPi) treatment [21,22,23] and reduce hyper-PARylation [18]. 

Due to the importance of PAR in the repair of DNA lesions and its relevance to emerging cancer therapies, the development of molecular tools to identify PAR formation and degradation have been of broad interest. In vitro PAR tools, including PAR antibodies such as the 10H mouse monoclonal antibody [24] and recent recombinant Fc fusions using different PBDs [25], can detect PAR in cell lysates or fixed cells but do not capture the dynamics of PAR formation and degradation in live cells. In vivo tools for PAR formation and accumulation include split luciferase fusions with the PBD of APLF [26] or bimolecular fluorescent complementation sensors using the PAR binding zinc finger (PBZ) domain from APLF or CHFR [27]. However, these probes rely on the use of the PBZ PBD, which we have shown offers a significantly lower dynamic range for PAR detection than the WWE domain from Ring Finger Protein 146 (RNF146) in micro-irradiation experiments [5]. Finally, the recent development of a FRET/FLIM assay based on the WWE domain from RNF146 provided a low but statistically significant FRET signal [28], suggesting the WWE domain may be useful in other in vivo assays to quantitate PAR formation.

We recently described the development of LivePAR (a genetically encoded fusion of the WWE domain from RNF146 to EGFP) to measure PAR formation at the site of laser micro-irradiation, demonstrating greater recruitment intensity when compared to all other PBDs tested [5]. Due to the limitations of current approaches for live cell PAR quantitation, we developed improved tools for the detection of PAR in cells using this WWE domain. First, we demonstrate that genotoxic treatment leads to an increase in nuclear enrichment of LivePAR that can be quantified by time-lapse microscopic imaging. This enables quantitation of PAR changes in single cells over time. Second, we generated split luciferase fusions of the WWE domain, developing a plate-based assay for PAR formation analysis and quantification. Here, we demonstrate each method reliably detects PAR at low μM doses of hydrogen peroxide (H_2_O_2_) or methyl methanesulfonate (MMS). Each method identifies hyper-PARylation in BRCA2 mutant cells that is eliminated following BRCA2 reversion. Finally, we demonstrate the application of the WWE split luciferase assay to characterize exposure to genotoxins, PARPi, and PARG inhibitors (PARGi).

## 2. Materials and Methods

### 2.1. Reagents and Chemicals

All plasmids, chemicals, and other reagents used in this study are listed in Supplement Table S1.

### 2.2. Cells and Cell Culture

A549 and U2OS cells were obtained from ATCC. BRCA2-deficient PEO1 cells and cisplatin-resistant BRCA2-revertant C4-2 cells were generous gifts from Sharon Cantor and Toshi Taniguchi [21,29]. All cells were cultured in DMEM supplemented with 10% heat-inactivated fetal bovine serum, L-glutamine, and penicillin/streptomycin. All parental and modified cell lines were cultured in tissue culture incubators at 37 °C, 5% CO_2_.

### 2.3. Lentivirus Production and Cell Transduction

Lentiviral particles were generated by co-transfection of 4 plasmids into 293-FT cells using the TransIT-X2 Transfection reagent: the packaging vectors pMD2.g (VSVG), pVSV-REV and pMDLg/pRRE together with the appropriate shuttle vectors. After 48 h, lentivirus-containing supernatant was collected and passed through 0.45 µM filters to isolate the viral particles as described previously [30,31].

Lentiviral transduction was performed as follows: cells (1 to 2 × 10^5^) were seeded into 6-well plates. After 24 h, lentiviral particles (1 mL) were mixed with polybrene (2 µg/mL) and added to the cells. Cells were incubated at 32 °C overnight, and then medium with lentiviral particles was removed and replaced with fresh medium. When cells were created to form stable cell lines, cells were then cultured for 48 h at 37 °C before selection with antibiotics (puromycin or hygromycin) for 1–2 weeks. When cells were transduced a second time to create a cell line expressing a fluorescently tagged fusion protein in addition to harboring a CRISPR/Cas9 vector, selection for the first stable cell line was completed and verified prior to initiation of the second transduction. When cells were created (transduced) for transient expression experiments, cells were cultured for at least 96 h, but no more than two weeks, at 37 °C before experimental analysis. 

### 2.4. CRISPR/Cas9-Mediated Knockout Cell Lines

A549 cell lines with CRISPR/Cas9-mediated depletion of BRCA2 were generated using the one vector CRISPR/Cas9 system (plentiCRISPR-v2; to deliver hSpcas9, gRNA and puromycin resistance). The plentiCRISPR-v2 vector was obtained from Addgene (plasmid #52961). The plentiCRISPR-v2 vectors containing control gRNA (5′-GCGTACCACACCCGTCGCAT), BRCA2g1 (5′-GAAACCATCTTATAATCAGC), and BRCA2-g2 (5′-TCGCACAGTGAAAACTAAAA) were gifts from Wim Vermeulen (Erasmus MC, The Netherlands) [32]. The plasmids were used to generate lentivirus for expression of Cas9 + Control-gRNA or Cas9 + BRCA2KO-gRNA, and A549 cells were transduced with lentivirus as indicated above. Cells were maintained in media containing puromycin (1 μg/mL) for 16 days, plated to generate single cell derived colonies, and validated by protein immunoblot to confirm BRCA2 depletion. Details of the technique have been described by us previously [33] and earlier by others [34].

### 2.5. Cell Protein Extract Preparation and Immunoblot

Protein extracts (whole cell lysates, WCL) were prepared from cells with different genetic modifications and/or different treatments as indicated in the text. Cells were seeded into a 60-mm cell culture dish. After reaching 75–80% confluency, cells were washed twice with cold PBS, collected, and lysed with an appropriate volume of 2× clear Laemmli buffer (2% SDS, 20% glycerol, 62.5 mmol/L Tris-HCl pH 6.8). Cell lysates were boiled for 10 min and quantified with the DC protein assay kit following the microplate protocol provided by the company (Bio-Rad; Hercules, CA, USA). For A549/BRCA2-deficient cell lysates, the same protocol was followed except cell lysates were not boiled but were lysed while on ice for 30 min with occasional pipetting to break cell membranes.

Whole cell protein lysates (15–40 μg protein) were loaded onto precast NuPAGE 4–12% Bis-Tris gels or, for BRCA2 immunoblots, onto precast NuPAGE^®^ 3–8% Tris-Acetate gels run 1 h at 120 V. Gel electrophoresis separated proteins were transferred onto a PVDF membrane or nitrocellulose membrane using a Turboblotter (Bio-Rad; Hercules, CA, USA). The membrane was first blocked with B-TBST (TBS buffer with 0.05% Tween-20 and supplemented with 5% blotting grade non-fat dry milk; Bio-Rad; Hercules, CA, USA) for 1 h at room temperature and subsequently blotted with the primary antibodies in B-TBST overnight at 4 °C. The primary antibodies and their dilutions are listed in Supplement Table S1. After washing, membranes were incubated with secondary antibodies in B-TBST for 1 h (room temperature). The following HRP conjugated secondary antibodies were used: Bio-Rad Goat anti-mouse-HRP conjugate and Bio-Rad anti-rabbit-HRP conjugate (see Supplement Table S1). After washing, the membrane was illuminated with a chemiluminescent substrate. Protein bands were imaged using a Bio-Rad Chemi-Doc MP imaging system.

### 2.6. Characterization of LivePAR Distribution

A549/LivePAR cells were seeded into each well of an 8-chamber glass bottom vessel (Thermo Fisher Scientific; Waltham, MA, USA; Cat# 155409) at a density of 5 × 10^4^ cells per well. After 24 h, cells were washed twice with 1XPBS, then fixed with 4% formaldehyde while simultaneously staining with Alexa Fluor 647 phalloidin (Invitrogen; Waltham, MA, USA; Cat# A22287) and NucBlue Fixed ReadyProbes reagent (Invitrogen; Waltham, MA, USA; Cat# R37606) for 30 min at 4 °C according to the manufacturer protocols. Cells were washed three times with 1XPBS, and cells were imaged using a Nikon A1rsi laser scanning confocal microscope.

### 2.7. Nuclear Enrichment of LivePAR

LivePAR expressing cells were seeded into each well of an 8-chamber glass bottom vessel (Thermo Fisher Scientific; Waltham, MA, USA; Cat# 155409) at a density of 5 × 10^4^ cells per well. After 24 h, cells were labeled with NucBlue Live ReadyProbes reagent (Invitrogen; Waltham, MA, USA; Cat# R37605) for 20 min at 37 °C, and then washed twice with fresh media. For experiments using PARPi (ABT-888; 10 μM) or PARGi (PDD00017273; 10 μM), cells were treated with the compounds for 1 h prior to adding NucBlue Live ReadyProbes reagent. Cells were imaged using a Nikon A1rsi laser scanning confocal microscope equipped with 6 visible wavelength lasers (405, 441, 514, 561, 647 nm, coherent) and a live-cell incubation chamber (Tokai Hit; Shizuoka, Japan) maintained at 5% CO_2_ and 37 °C, using a 20× (NA = 0.8) non-immersion objective. An image was taken before the genotoxic challenge, and subsequent time-lapse images were taken following the addition of the genotoxin for up to 30 min. Cell tracking and calculation of nuclear fluorescent intensities were performed in Nikon Elements. The intensity of nuclear LivePAR at each time point after genotoxin addition was normalized to the intensity of nuclear LivePAR prior to genotoxin addition, and normalization was performed for each cell individually. Cells were excluded from analysis if: (1) the mean intensity of fluorescent LivePAR signal in the nucleus was below a reliable threshold for measurement (i.e., too faint); (2) the mean intensity of fluorescent LivePAR signal in the nucleus was too high to obtain accurate dynamic changes (i.e., too bright); or (3) the cell moved during imaging and was absent from at least one of the imaging times. 

### 2.8. PAR Detection Using WWE Split Luciferase

For luminescent measurements, WWE split luciferase expressing cells were plated into 96 well plates (Thermo Fisher Scientific; Waltham, MA, USA; Cat# 136102) at a density of 5000–20,000 cells per well. After 24 h, cells were treated with compounds at the noted doses and durations, with either an equivalent volume of DMSO or normal media serving as a control. Following treatment, the media was removed and new media containing D-luciferin (150 µg/mL final concentration) was added for 4 min. Cells were then imaged using a BioTek Cytation 7 cell imaging multimode reader (Agilent; Santa Clara, CA, USA) using the associated Gen5 software. For each experiment, the luminescent intensity of a sample was normalized to untreated controls and reported as fold change of control.

### 2.9. PAR ELISA

PAR was measured using the HT PARP in vivo Pharmacodynamic ELISA Kit II (Bio-Techne; Minneapolis, MN, USA) as per the manufacturer instructions. U2OS cells were plated into 6 well dishes at a density of 5 × 10^5^ cells per well. After 24 h, cell medium was aspirated and replaced with fresh media containing MMS at the desired concentration or regular medium as control. For PARGi-treated samples, cells were pre-treated with PDD00017273 (10 μM) for 1 h prior to MMS treatment. Cells were treated for 30 min with MMS, after which cells were lysed on ice using the Cell Lysis Reagent, then scraped and collected into Eppendorf tubes. Samples were processed following manufacturer instructions and measured using a BioTek Cytation 7 imaging multimode reader (Agilent; Santa Clara, CA, USA) using the associated Gen5 software. PAR measurements were quantitated using standard curve samples provided. 

### 2.10. Laser Micro-Irradiation

For laser micro-irradiation, 5 × 10^4^ cells were seeded into each well of an 8-chamber glass bottom vessel (Thermo Fisher Scientific; Waltham, MA, USA; Cat# 155409). After 24 h, laser micro-irradiation and subsequent time-lapse imaging were performed using a Nikon A1rsi laser scanning confocal microscope equipped with 6 visible wavelength lasers (405, 441, 514, 561, 647 nm, Coherent), customized to add a UVA 355 nm laser (PicoQuant; Berlin, Germany) controlled by a Bruker XY Galvanometer, and equipped with a live-cell incubation chamber (Tokai Hit; Shizuoka, Japan) maintained at 5% CO_2_ and 37 °C, using a 20× (NA = 0.8) non-immersion objective for 405 nm laser micro-irradiation. For experiments using PARPi (ABT-888; 10 μM) or PARGi (PDD00017273; 10 μM), cells were treated with the compounds for 1 h prior to micro-irradiation. Micro-irradiation was performed at 100% laser power with a stimulation time of 0.125 s per site. For parallel irradiation, time lapse images were collected every 15 s during a 20 min interval. Images of focal recruitment were quantified using MIDAS for quantitation of and statistical analysis of focal recruitment [5]. Forty individual cells (2 sets of 10 cells were performed on 2 separate days) were analyzed and used to generate recruitment profiles and kinetic parameters.

### 2.11. CometChip Analysis

DNA damage analysis was performed as we described previously [35]. The 30-micron CometChips (glass-backed CometChip cassettes with 1 mm agarose and micro-patterned micro-wells each at 30-micron width), a well-former for 96-well assembly, the CometChip Electrophoresis System (CES) and the Comet Analysis Software (CAS) are available from BioTechne (Minneapolis, MN, USA). U2OS cells were plated into 96 well dishes at a density of 1 × 10^5^ cells per well. After 24 h, cell medium was aspirated and replaced with fresh media containing H_2_O_2_ or MMS at the desired concentration or regular medium as control. Following treatment for 30 min, cells were trypsinized and loaded into each well of a CometChip and kept at 4 °C for 30 min to allow the cells to settle into the microwells. Next, the CometChip was washed twice with 1XPBS and sealed with low melting point agarose (LMPA; Topvision; Thermo Fisher Scientific (Waltham, MA, USA); 7 mL; 0.75% LMPA/PBS). The CometChip was then submerged in lysis solution with detergent (BioTechne; Minneapolis, MN, USA) overnight at 4 °C. Electrophoresis of the CometChip was run under alkaline conditions (pH > 13; 200 mM NaOH, 1 mM EDTA, 0.1% Triton X-100) at 22 V for 50 min at 4 °C. After the electrophoresis step, the CometChip was re-equilibrated to neutral pH using two washings with Tris buffer (0.4 M Tris·Cl, pH 7.4 and 20 mM Tris·Cl, pH 7.4). The DNA was stained with 1XSYBR Gold dye (Thermo Fisher Scientific; Waltham, MA, USA) diluted in Tris buffer (20 mM Tris·Cl, pH 7.4) for 30 min and de-stained for 1 h in Tris buffer (20 mM Tris·Cl, pH 7.4). Comet images were collected using a Celigo imaging cytometer (Nexcelom Bioscience; Lawrence, MA, USA) at 1 μM/pixel resolution. The comets and the indicated DNA damage were then analyzed using the dedicated comet analysis software (CAS), and the data were exported to Excel (Microsoft; Redmond, WA, USA) and subsequently to Prism 9 (GraphPad Prism; San Diego, CA, USA) for statistical analysis. DNA damage is represented as % Tail DNA.

### 2.12. Statistical Analysis

Averages and standard error of the mean (SEM) were calculated from the means (on technical replicates) of multiple independent experiments (*n* = number of independent experiments as indicated in the figure legends) unless stated otherwise. Student *t*-test or ANOVA (with a Tukey post hoc test) was used to test for significant differences as appropriate, with results generally compared to controls and as indicated in the figure legends. In cases where comparisons are made to a different experimental group besides the control, it is stated in the figure legend. *p*-values are indicated by asterisks (* *p* < 0.05, ** *p* < 0.01, *** *p* < 0.001, **** *p* < 0.0001) or as stated in the figure legend. Statistical analyses were performed using GraphPad Prism v9 (San Diego, CA, USA) except those explicitly determined in MIDAS.

## 3. Results

### 3.1. LivePAR as a Tool for Live-Cell PAR Measurement

**Figure 1:**
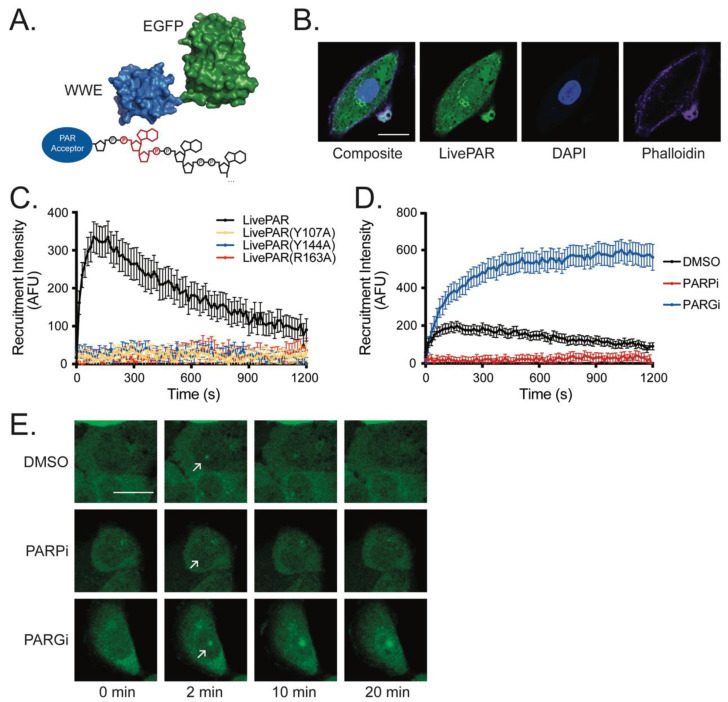
LivePAR as a molecular probe to investigate PARylation in live cells. (**A**) LivePAR is a fusion of the PAR-binding WWE domain from RNF146 (blue; PDB 3V3L) and EGFP (green; PDB 2Y0G) that preferentially binds to the iso-ADP-ribose moiety (red) of PAR chains [36,40]. (**B**) Expression of LivePAR in A549 cells. White scale bar denotes 20 µm. (**C**) Recruitment of LivePAR to sites of laser micro-irradiation (405 nm laser). Mutations in amino acids that eliminate PAR binding in the WWE domain (Y107A, Y144A, or R163A) prevent LivePAR recruitment; N ≥ 40 cells. (**D**) LivePAR recruitment to sites of laser-induced DNA damage is attenuated following PARPi treatment, while PARGi enhances and prolongs LivePAR recruitment; N ≥ 40 cells. (**E**) Images of LivePAR-expressing A549 cells following laser micro-irradiation. PARPi prevents foci formation while PARGi strengthens and prolongs foci formation compared to control. White scale bar denotes 20 µm.

We previously investigated the expression of PAR binding domains fused to EGFP as a tool for measuring PAR formation at sites of laser-induced DNA damage in live cells [5]. We investigated recruitment dynamics for 11 PAR binding domains, each encoding a fused C-terminal EGFP, following laser micro-irradiation [5]. The best performance was observed with the WWE domain from RNF146 fused to EGFP, which we termed LivePAR (Figure 1A). Expression of LivePAR is evenly distributed throughout the cell nucleus and cytoplasm (Figure 1B), and it readily recruits to laser micro-irradiation stimulation to which other PAR-dependent recruitment fusion proteins such as XRCC1-EGFP recruit [5]. Previous reports identified three critical amino acid residues in the WWE domain of RNF146 that facilitate PAR binding: Y107, Y144, and R163 [36]. Mutations in any one of these three amino acids prevented LivePAR recruitment to laser-induced DNA damage (Figure 1C). Pre-treatment of LivePAR-expressing cells with a PARPi (ABT-888) [37] prevented LivePAR recruitment by inhibiting PAR formation, while pre-treatment with a PARGi (PDD00017273) [38] prevented LivePAR dissociation from foci by inhibiting PAR degradation (Figure 1D,E). These data support our contention that LivePAR is recruiting to PAR formed at genomic sites following laser-induced DNA damage.

### 3.2. Development of the LivePAR Nuclear Enrichment Assay

**Figure 2:**
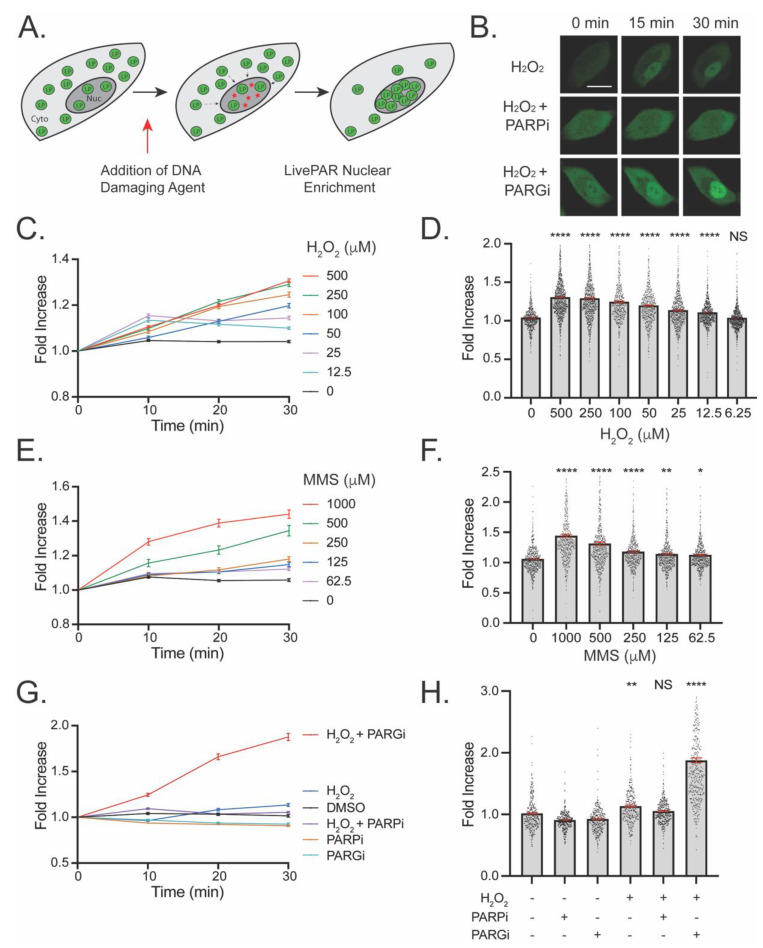
Detection of PARylation using LivePAR nuclear enrichment in live cells. (**A**) Diagram of mechanism: Prior to stress, LivePAR (green circles) is uniformly observed throughout a cell. H_2_O_2_ generates DNA damage in the nucleus (red stars) and stimulates PAR formation and LivePAR nuclear enrichment. (**B**) LivePAR nuclear enrichment following H_2_O_2_ (250 µM) exposure. PARPi (ABT-888; 10 µM) prevented LivePAR nuclear enrichment, while PARGi (PDD00017273; 10 µM) enhanced LivePAR nuclear enrichment. White scale bar denotes 20 µm. (**C**) Time course of LivePAR nuclear enrichment following H_2_O_2_ dose–response over 30 min. Graph shows mean ± SEM. (**D**) LivePAR nuclear enrichment 30 min following H_2_O_2_ dose–response. Each point represents a single cell nucleus. Graph shows mean ± SEM (in red). (**E**) Time course of LivePAR nuclear enrichment following MMS dose–response. Graph shows mean ± SEM. (**F**) LivePAR nuclear enrichment 30 min following MMS dose–response. Each point represents a single cell nucleus. Graph shows mean ± SEM (in red). (**G**) Time course of LivePAR nuclear enrichment following H_2_O_2_ (250 µM) treatment, with and without PARPi or PARGi. Graph shows mean ± SEM. (**H**) LivePAR nuclear enrichment 30 min following H_2_O_2_ (250 µM) treatment, with and without PARPi or PARGi. Each point represents a single cell nucleus. Graph shows mean ± SEM (in red). NS = no significance, * *p* < 0.05, ** *p* < 0.01, **** *p* < 0.0001; a two-sample t-test (compared to the untreated controls) for panels D and F, and a one-way ANOVA with Tukey post-hoc test (significance compared to untreated controls) for panel H. For panels C-H, each data point represents the mean ± SEM for at least 100 individual nuclei.

Although the LivePAR probe was originally designed to monitor PAR formed in response to laser-induced DNA damage, we next investigated the feasibility of using LivePAR to measure PAR formation following more broadly administered genotoxins such as H_2_O_2_ and MMS, both of which generate DNA damage repaired using PAR-mediated BER [2,39]. In preliminary tests, we observed that treatment of cells with H_2_O_2_ elicited nuclear enrichment of LivePAR that could be quantified using time-lapse imaging (Figure 2A,B). By treating cells with a nuclear-specific dye prior to genotoxic challenge, we were able to quantify the nuclear enrichment of LivePAR on a single cell basis over time (Supplement Figure S1A). Time-lapse images of LivePAR expressing cells demonstrated a dose-dependent response to H_2_O_2_ over a 30 min duration (Figure 2C). Quantitation revealed LivePAR nuclear enrichment analysis could detect H_2_O_2_-induced PAR formation in a dose-dependent manner down to 12.5 µM (Figure 2D). Similar time-lapse images and quantitation was performed following treatment with MMS over 30 min (Figure 2E), with LivePAR nuclear enrichment detecting MMS-induced PAR formation in a dose dependent manner, as low as 62.5 µM (Figure 2F). To validate that LivePAR nuclear enrichment is PAR-mediated, cells were pre-treated with either a PARPi (ABT-888) or a PARGi (PDD00017273), and cells were imaged for 30 min (Figure 2G). LivePAR nuclear enrichment could be prevented by PARPi pre-treatment or enhanced by PARGi pre-treatment (Figure 2H). Finally, we tested the nuclear enrichment of the mutant LivePAR(R163A) (a nonbinding fusion) to H_2_O_2_-induced PAR formation (Supplement Figure S1B). We observed no change in cellular distribution of LivePAR(R163A) following H_2_O_2_ treatment (Supplement Figure S1C). These results document that LivePAR nuclear enrichment can quantitatively assess PAR formation in live cells following genotoxic insult.

### 3.3. LivePAR Nuclear Enrichment to Detect BRCA2 Deficiency

**Figure 3:**
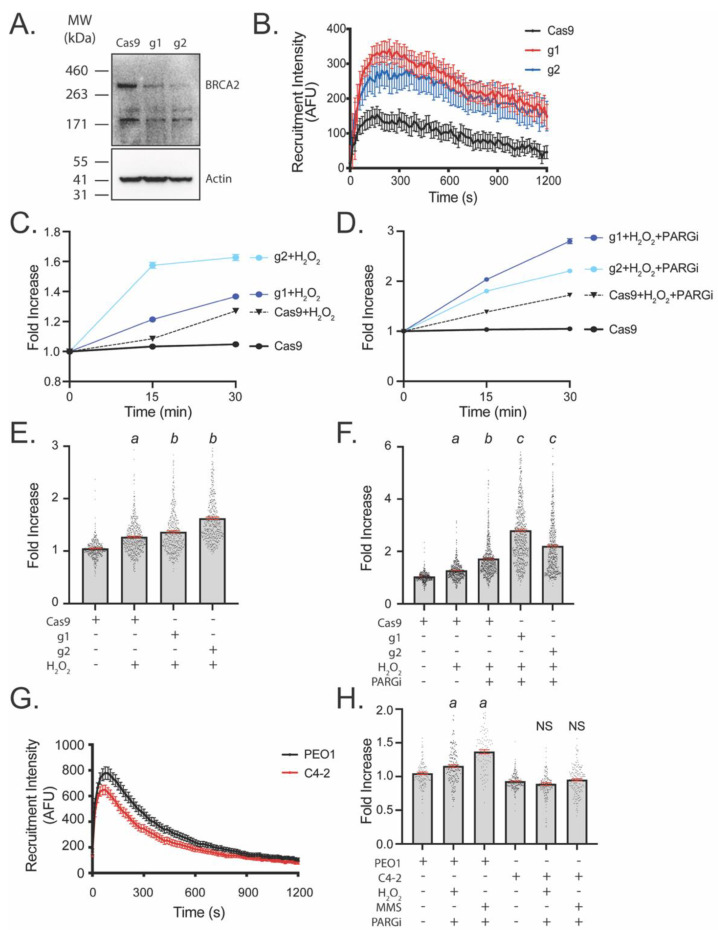
Enhanced PARylation in BRCA2-deficient cell lines. (**A**) Validation of A549/BRCA2-deficient cell lines. CRISPR/Cas9-mediated BRCA2 deficiencies were generated using 2 unique gRNAs (i.e., g1, g2). (**B**) Laser micro-irradiation of A549/Cas9 or A549/BRCA2-deficient cells; N ≥ 40 cells. (**C**) Time course of LivePAR nuclear enrichment in BRCA2-deficient cells following H_2_O_2_ (250 µM) dose–response over 30 min. (**D**) Time course of LivePAR nuclear enrichment in BRCA2-deficient cells following H_2_O_2_ dose–response over 30 min in PARGi pretreated cells. (**E**) LivePAR nuclear enrichment 30 min following H_2_O_2_ dose–response in BRCA2-deficient cells. Each point represents a single cell nucleus. Graph shows mean ± SEM (in red). (**F**) LivePAR nuclear enrichment with 1 h PARGi pre-treatment followed by 30 min H_2_O_2_ exposure in BRCA2-deficient cells. Each point represents a single cell nucleus. Graph shows mean ± SEM (in red). (**G**) Laser micro-irradiation of PEO1/LivePAR or C4-2/LivePAR cells; N ≥ 40 cells. Graph shows mean ± SEM. (**H**) LivePAR nuclear enrichment in PEO1 or C4-2 cells following 1 h PARGi pre-treatment and 30 min H_2_O_2_ exposure. Each point represents a single cell nucleus. Graph shows mean ± SEM (in red). For statistical comparisons from ANOVA with Tukey post hoc test: a: *p* < 0.01 in comparison to Cas9 or untreated controls; b: *p* < 0.01 in comparison to Cas9-H_2_O_2_ cells; c: *p* < 0.01 in comparison to Cas9-H_2_O_2_-PARGi cells; NS = no significance.

BRCA2 deficiency is associated with hyperactivated PARP1, while reversion and reactivation of BRCA2 following cisplatin therapy is associated with decreased PAR and increased PARPi resistance [18,20,22,23]. We used two different approaches to determine if the LivePAR nuclear enrichment assay could be used to identify basal PAR changes due to BRCA2 status. First, a CRISPR/Cas9-mediated BRCA2 deficiency was generated in A549 cells, which harbor no known mutations/deficiencies in homologous recombination or PAR-mediated single-strand break repair. Two different cells lines were created using two unique gRNAs, with each showing a decrease in the expression of BRCA2 (Figure 3A). The A549/BRCA2-deficient cell lines showed increased LivePAR recruitment following laser micro-irradiation, indicative of increased PARylation (Figure 3B). Further, H_2_O_2_ treatment increased LivePAR nuclear enrichment in both BRCA2 deficient cell lines (Figure 3C), while pre-treatment with PARGi amplified the intensity of the LivePAR nuclear enrichment observed following H_2_O_2_ treatment (Figure 3D). H_2_O_2_ treatment elicited a 37% increase in LivePAR nuclear enrichment in the A549/BRCA2-g1 cell line, a 63% increase in the A549/BRCA2-g2 cell line, and only a 27% increase in H_2_O_2_ treated A549/Cas9 cells, when compared to untreated control cells (Figure 3E). When pretreating cells with PARGi, H_2_O_2_ treatment elicited a 180% increase in LivePAR nuclear enrichment in the A549/BRCA2-g1 cell line, a 121% increase in the A549/BRCA2-g2 cell line, and only a 72% increase in A549/Cas9 cells, when compared to untreated control cells (Figure 3F). Overall, we find that BRCA2 deficiency generates a significant increase in PARylation that was detectable using the LivePAR nuclear enrichment assay.

To determine if the LivePAR nuclear enrichment assay could identify decreased PARylation associated with cisplatin-mediated reversion and reactivation of BRCA2, we compared the BRCA2 deficient ovarian adenocarcinoma cell line PEO1 with its cisplatin-resistant BRCA2-revertant clone C4-2 [21]. PEO1 cells show no detectable expression of full length BRCA2 whereas C4-2 clones were derived from PEO1 following 4 weeks of cisplatin treatment and show restored expression [21]. C4-2 cells demonstrated a decrease in LivePAR foci intensity following laser micro-irradiation when compared to PEO1, suggesting a decrease in PARylation in the BRCA2-revertant C4-2 cells (Figure 3G). LivePAR nuclear enrichment was detectable in PEO1 cells following H_2_O_2_ + PARGi treatment or MMS + PARGi treatment when compared to untreated controls, while no significant change in LivePAR nuclear enrichment was observed following similar conditions in C4-2 cells (Figure 3H). These results support our conclusion that the LivePAR nuclear enrichment assay is able to identify PARylation changes associated with BRCA2 deficiency and BRCA2 reversion.

### 3.4. Development of WWE-Mediated Split Luciferase PAR Detection Assay

**Figure 4:**
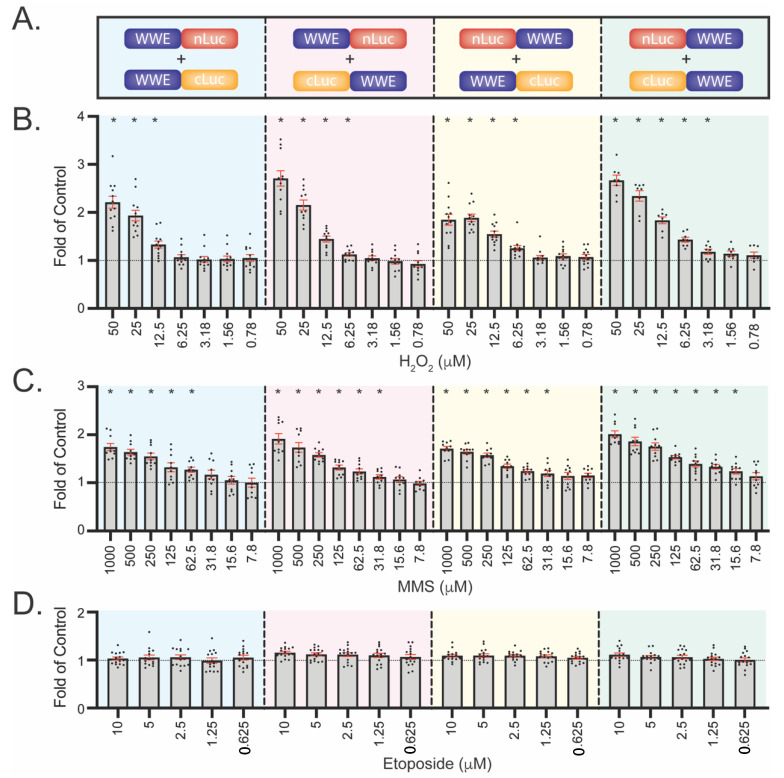
Development of WWE split luciferase (Luc) assay to measure PARylation. (**A**) Vector designs to assess the effect of WWE and split-Luc orientation. WWE was fused to either the N-terminal side of nLuc or cLuc, or to the C-terminal side of nLuc or cLuc. Four combinations were tested. (**B**) PAR formation following 30 min H_2_O_2_ dose–response. Values are normalized to untreated or DMSO controls. (**C**) PAR formation following 30 min MMS dose–response. (**D**) PAR formation following 30 min etoposide dose–response. Each dot represents a single plate reading, with mean and SEM (red bars) shown; N ≥ 8 reads. * *p* < 0.05 using a one-sample *t*-test.

While the LivePAR nuclear enrichment assay enables cell level analysis of PAR formation, it has two drawbacks: (1) background levels of LivePAR expression could mask fluorescent increases in nuclear localization by reducing the dynamic range of imaging; and (2) collection of time-lapse fluorescent images of this nature requires a confocal microscope. To address these issues, we developed a plate-based luminescence assay to measure PAR formation using split luciferase probes designed with a WWE domain fused to either the N-terminal portion of luciferase (nLuc) or the C-terminal portion of luciferase (cLuc). As the orientation of the WWE and nLuc or cLuc fusion could reduce signal intensity, we tested all four combination pairs (Figure 4A). First, U2OS cells were transduced using lentiviral vectors expressing the individual WWE-nLuc proteins to create stable cell lines. Next, the U2OS/WWE-nLuc cells were subsequently transduced using lentiviral vectors for expression of the WWE-cLuc proteins (Supplement Figure S2A).

We observed luminescence only in the U2OS cells expressing both the nLuc and cLuc fusions (Supplement Figure S2B). Treatment with H_2_O_2_ elicited a dose-dependent response for each of the four WWE-nLuc/WWE-cLuc pairs (up to 50 µM H_2_O_2_) with varying sensitivity; concentrations above 50 µM H_2_O_2_ produced interference with the luciferase signal in U2OS cells (Figure 4B). While all of the WWE-nLuc/WWE-cLuc pairs were sensitive enough to detect PAR formation following 12.5 µM H_2_O_2_ for 30 min, one of the pairs reported significant PAR formation in response to 3.18 µM H_2_O_2_ treatment. We observed a similar dose-dependent response in PAR formation following MMS, with all of the WWE-nLuc/WWE-cLuc pairs demonstrating significant PAR formation following 62.5 µM MMS and one pair sensitive to detect PAR down to 15.6 µM MMS (Figure 4C). When cells were treated with etoposide, a chemotherapeutic agent that does not induce PARP1 activation upon acute exposure, no observable PAR formation was observed (Figure 4D).

**Figure 5:**
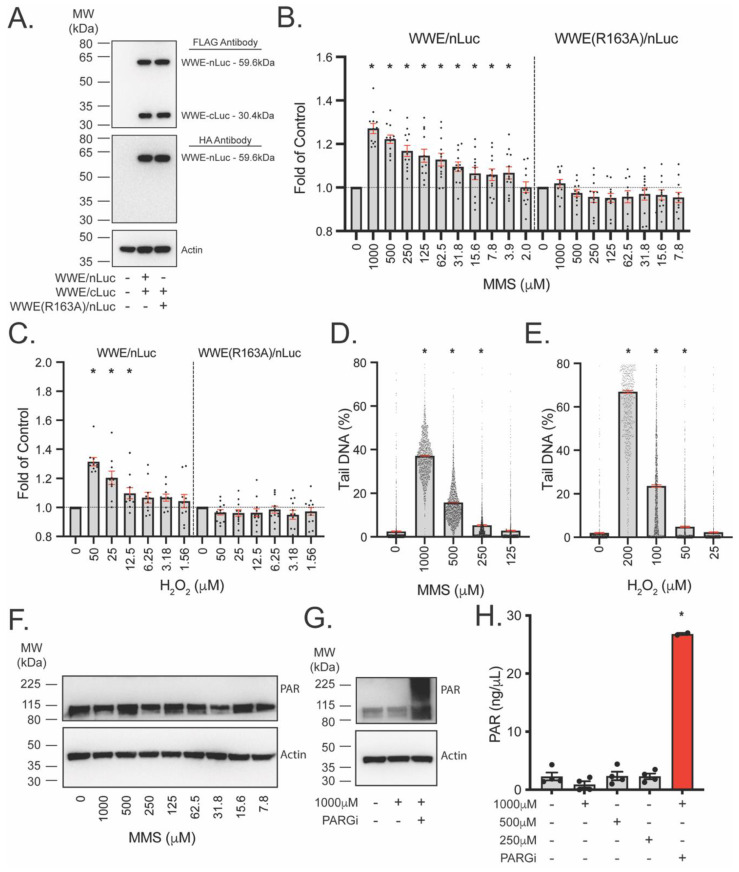
Sensitivity of the single vector WWE split luciferase (Luc) assay. (**A**) Expression of WWE-nLuc and WWE-cLuc in U2OS cells using a single lentiviral vector. WWE(R163A)-nLuc was alternatively expressed to control for luciferase binding effects and serve as a non-PAR binding control. (**B**) PAR formation following 30 min MMS dose–response. Values are normalized to untreated controls. (**C**) PAR formation following 30 min H_2_O_2_ dose–response. (**D**) CometChip results following 30 min MMS dose–response. (**E**) CometChip results following 30 min H_2_O_2_ dose–response. (**F**) Immunoblot for PAR formation in U2OS cells following 30 min MMS dose–response. (**G**) Immunoblot for PAR formation in U2OS cells following 30 min MMS (1000 μM) with or without PARGi pre-treatment (PDD00017273; 10 μM, 1 h). (**H**) PAR ELISA results following 30 min MMS (1000 μM) with or without PARGi pre-treatment (PDD00017273; 10 μM, 1 h). For (**B**,**C**), each dot represents a single plate reading, with mean and SEM (red bars) shown; N ≥ 8 reads. * *p* < 0.05 using a one-sample *t*-test. For (**D**,**E**), each dot represents a single cell, with mean and SEM (red bars) shown; * *p* < 0.05 using an ANOVA with Tukey post hoc test when compared to untreated control. For (**H**): * *p* < 0.05 using an ANOVA with Tukey post hoc test when compared to untreated control.

To better control for protein expression levels, we created a single vector with both WWE-nLuc and WWE-cLuc proteins encoded and linked by a T2A sequence [41]. Despite the higher sensitivity in PAR detection using the fusions with C-terminal WWEs, we chose to use the N-terminal WWE fused to C-terminal split luciferase components due to the overall higher level of luminescence in that pairing (Supplement Figure S2C). Using a single vector, we observed nearly equal expression of the WWE-nLuc and WWE-cLuc proteins (Figure 5A). Additionally, we developed a second vector that co-expressed the WWE-cLuc protein with a mutated, nonbinding WWE(R163A)-nLuc to control for luminescent intensity changes related to a compound or genetic modification not related to PAR formation. The single vector PAR binding pair showed significant PAR formation following 3.9 µM MMS (Figure 5B) and 12.5 µM H_2_O_2_ (Figure 5C). No luminescent signal change was observed with the WWE(R163A)-nLuc expressing cells, further supporting our conclusion that the increase in luminescence is a measure of PAR formation.

We compared the sensitivity of split luciferase PAR detection to other assays for the detection of PAR or DNA damage. Following comparable doses of MMS and H_2_O_2_ in U2OS cells, the CometChip assay, which detects DNA damage on a single-cell basis, was able to detect DNA damage at concentrations of 250 µM MMS (Figure 5D) and 50 µM H_2_O_2_ (Figure 5E), about 64X the MMS concentration and 4X the H_2_O_2_ concentration at which the WWE split luciferase assay detects PARylation. PAR immunoblots showed no detectable PAR following 30 min of any comparable dose of MMS in U2OS cells (Figure 5F). However, PAR could be detected following MMS (1 mM) when pretreated with a PARGi (Figure 5G). Finally, PAR ELISA results did not reflect significant changes in PARylation following 30 min MMS exposure without PARGi pre-treatment (Figure 5H). These results suggest that the WWE split luciferase PAR assay is more sensitive than the CometChip assay, PAR immunoblots, or PAR ELISAs in detecting PAR (or DNA damage to induce PAR) in cells.

### 3.5. WWE Split Luciferase Assay for Detecting BRCA2 Deficiency

**Figure 6:**
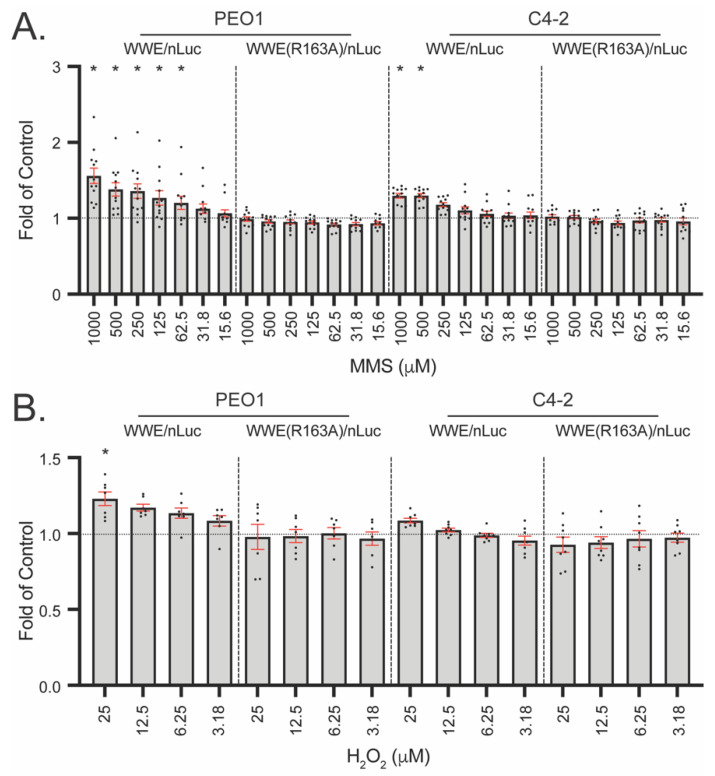
Enhanced PARylation in BRCA2-deficient cells identified using the WWE split luciferase assay. WWE-nLuc [or WWE(R163A)-nLuc] and WWE-cLuc were expressed in BRCA2-deficient PEO1 or BRCA2-resurgent C4-2 cells using a single lentiviral vector. (**A**) PAR formation following 30 min MMS dose–response. Values are normalized to untreated controls. (**B**) PAR formation following 30 min H_2_O_2_ dose–response. For (**A**,**B**), each dot represents a single plate reading, with mean and SEM (red bars) shown; N ≥ 6 reads. * *p* < 0.05 using a one-sample *t*-test.

As LivePAR nuclear enrichment was sensitive at detecting BRCA2-associated changes in PARylation (Figure 3), we investigated if the WWE split luciferase assay could also be used to identify similar BRCA2-reversions. WWE-nLuc (or nonbinding WWE(R163A)-nLuc controls) and WWE-cLuc were expressed in the BRCA2-deficient PEO1 cells and the BRCA2-revertant C4-2 cells. The PAR binding probe revealed significant PAR formation following 62.5 µM MMS (Figure 6A) and 25 µM H_2_O_2_ (Figure 6B) in PEO1 cells. In contrast, significant PAR formation was only observed in C4-2 cells following 500 µM MMS (Figure 6A) while no significant difference was observed with H_2_O_2_ (Figure 6B). These results suggest that the split luciferase assay is sensitive at detecting BRCA2-associated changes in PARylation independent of the damage type (i.e., oxidative, alkylative).

### 3.6. WWE Split Luciferase Assay as a Screening Tool

**Figure 7:**
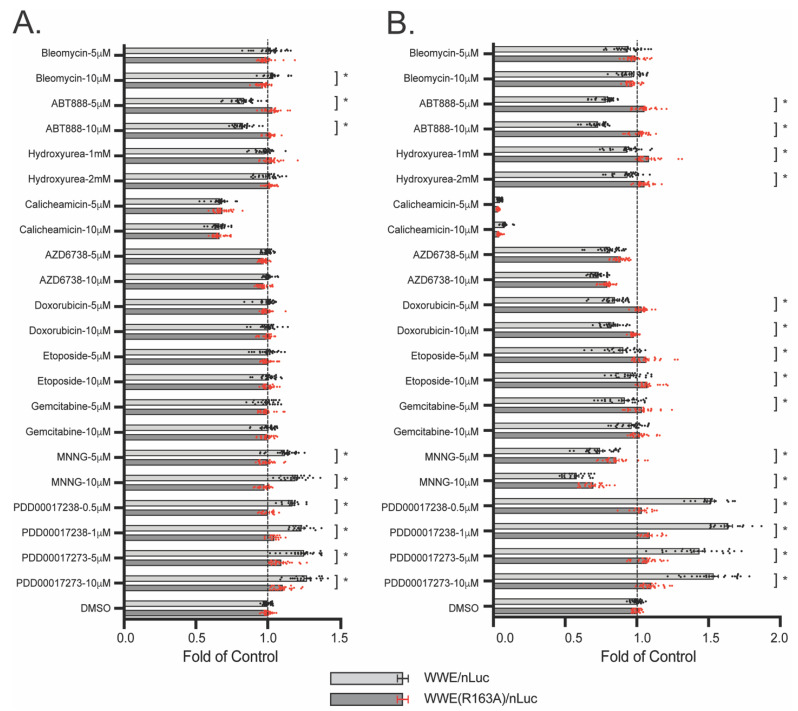
Genotoxin screening for PARylation using the WWE split luciferase assay. U2OS cells expressing WWE-nLuc (or WWE(R163A)-nLuc) and WWE-cLuc were treated with individual compounds at the specified concentration. The vertical dotted line denotes a DMSO-normalized value. (**A**) PAR formation following 30 min of compound exposure. Values are normalized to untreated controls. (**B**) PAR formation following 6 h of compound exposure. For (**A**,**B**), each dot represents a single plate reading, with mean and SEM (red bars) shown; N ≥ 8 reads. * *p* < 0.05 using a two-sample *t*-test in comparison between WWE-nLuc and WWE(R163A)-nLuc for each test condition.

Give the strength of the WWE split luciferase assay in evaluating cellular PAR formation in response to genotoxin exposure, we then tested a select set of chemotherapeutic and DNA damaging agents to identify changes in PAR formation following acute exposure (30 min) or a longer exposure time that will allow analysis of the impact on replication stress (6 h). No cell death is observed at these doses or exposure times. Compounds with mechanisms not impacting PARylation (such as bleomycin, hydroxyurea, ATR inhibitor AZD6738, doxorubicin, etoposide, and gemcitabine) showed no change in PAR following 30 min of exposure (Figure 7A). Conversely, the alkylating agent N-Methyl-N’-nitro-N-nitrosoguanidine (MNNG), and two PARGi (PDD00017273 and PDD00017238) showed an increase in PAR-associated luminescence. The PARPi ABT-888 attenuated PAR-associated luminescence, which was anticipated. Interestingly, calicheamicin reduced the overall luciferase signal in both the WWE-nLuc and the nonbinding WWE(R163A)-nLuc controls (Figure 7A), highlighting the importance of including the WWE(R163A)-nLuc probe to control for PAR-independent changes in luminescence, which could be misinterpreted without it.

When the exposure time was increased to 6 h, we confirmed continuing suppression of PAR luminescence in the ABT-888 PARPi samples (Figure 7B). We also observed minor decreases in PAR luminescence following treatment with hydroxyurea, doxorubicin, or etoposide when compared to WWE(R163A)-nLuc controls. Additionally, while both PARG inhibitors show sustained increases in PAR-associated luminescence, the DNA damaging agent MNNG showed a decrease in PAR at 6 h (Figure 7B). This suggests that PARGi can be differentiated from acute exposure to DNA damaging agents that induce PAR formation, based on the prolonged luminescence from PARGi treatment observed in the WWE split luciferase assay.

## 4. Discussion

PAR is important in the repair of DNA damage in multiple repair pathways including BER [6,10,42]. PAR synthesis and degradation have gained increasing cancer relevance due to the synthetic lethality observed between PARP inhibition and BRCA deficiencies in a variety of cancers [12,13]. Due to the role of PARylation in DNA damage and repair as well as its increasing importance in cancer progression and therapy, multiple molecular tools have been developed to measure PARylation. PAR antibodies such as the 10H mouse monoclonal antibody [24] and more recent recombinant Fc fusions using PAR binding domains [25] can be used only to detect in vitro PAR accumulation. In vivo tools for PAR formation and accumulation include bimolecular fluorescent complementation sensors [27] or split luciferase fusions with the PBD of APLF or CHFR [26]. Both of these probes rely on the use of the PBZ PAR binding domain, which we have shown offers a significantly lower dynamic range for PAR binding/recruitment than the WWE domain from RNF146 [5]. The use of fluorescent NAD^+^ analogues for two-color imaging and FRET/FLIM experiments to measure PAR formation [43,44], while accurate for temporal changes in PAR formation, may artificially increase PARylation by enhancing the available NAD^+^ substrate pool for PARylation in a manner similar to NAD^+^ augmenting compounds like dihydronicotinamide riboside (NRH) [5]. Considering these limitations, we sought to create an improved tool set for the detection of PAR in live cells using EGFP or split luciferase fusions of the PAR binding WWE domain from RNF146. Similar methods have used macrodomains [45], but we have shown the macrodomain from H2A1.1 offers a significantly lower dynamic range for PAR binding recruitment than the WWE domain from RNF146 [5], possibly due to lower PAR binding moiety availability. Here, we describe two unique approaches to quantify PAR formation using the WWE domain from RNF146.

Nuclear enrichment of LivePAR provides single-cell quantitation of PAR using time-lapse imaging. As DNA damage is generated, LivePAR concentrates in the nuclear compartment, increasing fluorescent (EGFP) intensity that can be measured using a nuclear counterstain in live cells (Figure 2A). Use of time-lapse images enables dynamic quantification of how rapidly PAR forms or how long a compound may generate PAR following initial exposure, and the only limit observed for experimental duration is the capability for reliable cell/nuclei tracking. We observed that nuclear enrichment of LivePAR was PAR dependent, as PARPi attenuated enrichment while PARGi strengthened the signal (Figure 2B). This effect was seen using H_2_O_2_ and MMS, genotoxins that generate DNA damage repaired via PAR-mediated pathways but differ in the type of DNA damage generated. LivePAR nuclear enrichment was sensitive to concentrations as low as 12.5 μM H_2_O_2_ and 62.5 μM MMS. This exceeded the sensitivity observed with either the CometChip assay, PAR immunoblots, or PAR ELISAs (Figure 5). The method is robust and provides quantitation of the temporal accumulation of PAR, but it relies on quantitation of time-lapse fluorescent images collected and analyzed for tracked single cell nuclei. However, as a visual field can contain hundreds of cells, LivePAR nuclear enrichment can be quantified for a substantial number of individual cells simultaneously.

The WWE split luciferase assay provides a plate-based approach for the quantitation of PAR that is equal to or more sensitive to the LivePAR nuclear enrichment assay. The orientation of the WWE PAR binding domain with respect to either nLuc or cLuc had a minor impact on normalized values following genotoxic stress, but the total luminescent intensity with the WWE domain fused to the N-terminus of either nLuc (i.e., WWE-nLuc) or cLuc (i.e., WWE-cLuc) yielded a greater signal and was chosen for the single vector design (Figure 4). When the WWE split luciferase single vector design was used, we observed better control of the stoichiometric ratio of WWE-nLuc and WWE-cLuc proteins (Figure 5A), and we observed PAR formation sensitivity to concentrations as low as 12.5 μM H_2_O_2_ and 3.9 μM MMS following 30 min exposure, which exceeded the sensitivity observed using the CometChip assay, PAR immunoblots, or PAR ELISAs (Figure 5).

One important finding was that at concentrations above 50 μM H_2_O_2_ in U2OS cells, we observed suppression of luminescence which we attributed to the oxidative effects of H_2_O_2_. Due to this observation, we utilized the WWE(R163A) mutation to control for luminescence changes mediated by compound effects on luciferase activity or protein stability. The importance of this control is demonstrated in the case of the enediyne antitumor antibiotic calicheamicin [46] where WWE-nLuc cells displayed decreased luminescence following treatment (Figure 7). As similar suppressive changes were observed in the WWE(R163A)-nLuc cells following calicheamicin, we classified calicheamicin as not affecting PAR formation but rather impacting luciferase activity or protein stability.

The WWE split luciferase approach is readily amenable to genotoxin screens and drug discovery workflows. We tested a select number of genotoxic compounds that were known to promote or alter PARylation (e.g., MNNG, ABT-888, PDD00017273) in addition to compounds that do not directly target PAR formation (e.g., etoposide, bleomycin, doxorubicin). The alkylating agent MNNG promoted PAR formation after 30 min of exposure, at levels like that seen with the PARGi PDD00017273 (Figure 7A). However, by 6 h, MNNG-mediated PAR effects began to diminish while PARGi effects were sustained (Figure 7B). The cause of the diminished luminescence observed in MNNG treated cells is unknown, but we hypothesize that the loss of PARylation can be due to completion of BER-mediated repair of the MNNG-induced DNA lesions or due to increased ubiquitylation and subsequent degradation of the PAR-bound WWE split luciferase probe following DNA damage. The presence of the WWE PAR binding domain in the E3 ligase RNF146 suggests the presence of at least one but likely more E3 ligases localized to PAR formed in response to DNA damage. The ubiquitylation of the WWE split luciferase fusion protein would lead to greater degradation of the PAR-bound WWE split luciferase probes. This effect would not be observed in the R163A control as it is not localized to PAR during DNA damage and therefore not in proximity to PAR-localized E3 ligases, and it would not be observed in the PARGi samples as the WWE split luciferase is not released from PAR to enable degradation. Similar sustained effects were observed with PARGi PDD00017238, which indicates that PARGi can be identified and differentially classified from acute DNA damaging agents using the WWE split luciferase approach. This is especially important as recent research points to successful use of PARGi in cancer treatment paradigms [47,48,49]. Additionally, PARPi such as ABT-888 can also be identified using the WWE split luciferase approach. Lastly, though we did not anticipate it, we observed a small but significant decrease in PARylation following 6 h doxorubicin and etoposide treatments (Figure 7B). The reason for this is unknown, but because both doxorubicin and etoposide are documented topoisomerase II inhibitors while bleomycin and AZD6738 (which showed no effect) are not, this may suggest a relationship where topoisomerase II activity negatively affects PAR levels. 

Both the nuclear enrichment assay and the WWE split luciferase assay reported enhanced PARylation in BRCA2-deficient cells. For CRISPR/Cas9-mediated BRCA2-modified A549 cells, we observed increased PARylation in BRCA2-depleted cells when compared to controls using the nuclear enrichment assay (Figure 3), suggesting BRCA2 depletion enhances the overall level of PARylation. Similarly, cisplatin-resistant, BRCA2-resurgent C4-2 cells demonstrated lower PARylation than BRCA2-deficient PEO1 cells by both the nuclear enrichment and the WWE split luciferase assays (Figure 3 and Figure 6). These results suggest that loss of BRCA2 leads to hyper-PARylation in cells and demonstrates that both assays may be used to identify BRCA2 deficiencies or reversion in cells by measuring PAR status.

## 5. Conclusions

Due to the importance of PARylation in managing critical pathways of the DNA damage response, identification of sensitive yet robust methods to quantitatively measure changes in PARylation in live cells remains an important research goal. Here, we provide two methods to measure PARylation following genotoxic challenge or genetic alterations. Nuclear enrichment of LivePAR provides a visual and quantitative method to assess PAR formation in a single cell, time-lapse manner. The WWE split luciferase approach provides a rapid, plate-based assay to measure PAR formation of multiple experimental conditions simultaneously. Both approaches can be used for genetic screening (e.g., BRCA2 deficiency or reversion) or compound screening (e.g., genotoxins, PARPi, or PARGi).

## Data Availability

All data reported in this paper will be shared by the lead contact upon request. Any additional information required to reanalyze the data reported in this paper is available from the lead contact upon request.

## Supplementary Materials

The following supporting information can be downloaded at: https://www.mdpi.com/article/10.3390/cancers14153676/s1, Figure S1: LivePAR nuclear enrichment assay; Figure S2: Comparison of WWE split luciferase (Luc) individual construct pairings; Figure S3: Uncropped immunoblot images for Figure 3A, Figure 5A,F,G; Table S1: Reagent List [50].

## References

[1] Hanahan D., Weinberg R.A. (2011). Hallmarks of cancer: The next generation. Cell.

[2] Almeida K.H., Sobol R.W. (2007). A unified view of base excision repair: Lesion-dependent protein complexes regulated by post-translational modification. DNA Repair.

[3] Krokan H.E., Bjoras M. (2013). Base excision repair. Cold Spring Harb. Perspect. Biol..

[4] Abbotts R., Wilson D.M. (2017). Coordination of DNA single strand break repair. Free Radic. Biol. Med..

[5] Koczor C.A., Saville K.M., Andrews J.F., Clark J., Fang Q., Li J., Al-Rahahleh R.Q., Ibrahim M., McClellan S., Makarov M.V. (2021). Temporal dynamics of base excision/single-strand break repair protein complex assembly/disassembly are modulated by the PARP/NAD(+)/SIRT6 axis. Cell Rep..

[6] El-Khamisy S.F., Masutani M., Suzuki H., Caldecott K.W. (2003). A requirement for PARP-1 for the assembly or stability of XRCC1 nuclear foci at sites of oxidative DNA damage. Nucleic Acids Res..

[7] Dantzer F., de La Rubia G., Menissier-De Murcia J., Hostomsky Z., de Murcia G., Schreiber V. (2000). Base excision repair is impaired in mammalian cells lacking Poly(ADP-ribose) polymerase-1. Biochemistry.

[8] Schreiber V., Ame J.C., Dolle P., Schultz I., Rinaldi B., Fraulob V., Menissier-de Murcia J., de Murcia G. (2002). Poly(ADP-ribose) polymerase-2 (PARP-2) is required for efficient base excision DNA repair in association with PARP-1 and XRCC1. J. Biol. Chem..

[9] Teloni F., Altmeyer M. (2016). Readers of poly(ADP-ribose): Designed to be fit for purpose. Nucleic Acids Res..

[10] Saville K.M., Clark J., Wilk A., Rogers G.D., Andrews J.F., Koczor C.A., Sobol R.W. (2020). NAD(+)-mediated regulation of mammalian base excision repair. DNA Repair.

[11] Fouquerel E., Sobol R.W. (2014). ARTD1 (PARP1) activation and NAD(+) in DNA repair and cell death. DNA Repair.

[12] Bryant H.E., Schultz N., Thomas H.D., Parker K.M., Flower D., Lopez E., Kyle S., Meuth M., Curtin N.J., Helleday T. (2005). Specific killing of BRCA2-deficient tumours with inhibitors of poly(ADP-ribose) polymerase. Nature.

[13] Farmer H., McCabe N., Lord C.J., Tutt A.N., Johnson D.A., Richardson T.B., Santarosa M., Dillon K.J., Hickson I., Knights C. (2005). Targeting the DNA repair defect in BRCA mutant cells as a therapeutic strategy. Nature.

[14] Cong K., Peng M., Kousholt A.N., Lee W.T.C., Lee S., Nayak S., Krais J., VanderVere-Carozza P.S., Pawelczak K.S., Calvo J. (2021). Replication gaps are a key determinant of PARP inhibitor synthetic lethality with BRCA deficiency. Mol. Cell.

[15] Paes Dias M., Tripathi V., van der Heijden I., Cong K., Manolika E.M., Bhin J., Gogola E., Galanos P., Annunziato S., Lieftink C. (2021). Loss of nuclear DNA ligase III reverts PARP inhibitor resistance in BRCA1/53BP1 double-deficient cells by exposing ssDNA gaps. Mol. Cell.

[16] Vaitsiankova A., Burdova K., Sobol M., Gautam A., Benada O., Hanzlikova H., Caldecott K.W. (2022). PARP inhibition impedes the maturation of nascent DNA strands during DNA replication. Nat. Struct. Mol. Biol..

[17] Hanzlikova H., Kalasova I., Demin A.A., Pennicott L.E., Cihlarova Z., Caldecott K.W. (2018). The Importance of Poly(ADP-Ribose) Polymerase as a Sensor of Unligated Okazaki Fragments during DNA Replication. Mol. Cell.

[18] Gottipati P., Vischioni B., Schultz N., Solomons J., Bryant H.E., Djureinovic T., Issaeva N., Sleeth K., Sharma R.A., Helleday T. (2010). Poly(ADP-ribose) polymerase is hyperactivated in homologous recombination-defective cells. Cancer Res..

[19] Panzarino N.J., Krais J.J., Cong K., Peng M., Mosqueda M., Nayak S.U., Bond S.M., Calvo J.A., Doshi M.B., Bere M. (2021). Replication Gaps Underlie BRCA Deficiency and Therapy Response. Cancer Res..

[20] Fong P.C., Boss D.S., Yap T.A., Tutt A., Wu P., Mergui-Roelvink M., Mortimer P., Swaisland H., Lau A., O’Connor M.J. (2009). Inhibition of poly(ADP-ribose) polymerase in tumors from BRCA mutation carriers. N. Engl. J. Med..

[21] Sakai W., Swisher E.M., Jacquemont C., Chandramohan K.V., Couch F.J., Langdon S.P., Wurz K., Higgins J., Villegas E., Taniguchi T. (2009). Functional restoration of BRCA2 protein by secondary BRCA2 mutations in BRCA2-mutated ovarian carcinoma. Cancer Res..

[22] Sakai W., Swisher E.M., Karlan B.Y., Agarwal M.K., Higgins J., Friedman C., Villegas E., Jacquemont C., Farrugia D.J., Couch F.J. (2008). Secondary mutations as a mechanism of cisplatin resistance in BRCA2-mutated cancers. Nature.

[23] Edwards S.L., Brough R., Lord C.J., Natrajan R., Vatcheva R., Levine D.A., Boyd J., Reis-Filho J.S., Ashworth A. (2008). Resistance to therapy caused by intragenic deletion in BRCA2. Nature.

[24] Kawamitsu H., Hoshino H., Okada H., Miwa M., Momoi H., Sugimura T. (1984). Monoclonal antibodies to poly(adenosine diphosphate ribose) recognize different structures. Biochemistry.

[25] Gibson B.A., Conrad L.B., Huang D., Kraus W.L. (2017). Generation and Characterization of Recombinant Antibody-like ADP-Ribose Binding Proteins. Biochemistry.

[26] Furman J.L., Mok P.W., Shen S., Stains C.I., Ghosh I. (2011). A turn-on split-luciferase sensor for the direct detection of poly(ADP-ribose) as a marker for DNA repair and cell death. Chem. Commun..

[27] Krastev D.B., Pettitt S.J., Campbell J., Song F., Tanos B.E., Stoynov S.S., Ashworth A., Lord C.J. (2018). Coupling bimolecular PARylation biosensors with genetic screens to identify PARylation targets. Nat. Commun..

[28] Serebrovskaya E.O., Podvalnaya N.M., Dudenkova V.V., Efremova A.S., Gurskaya N.G., Gorbachev D.A., Luzhin A.V., Kantidze O.L., Zagaynova E.V., Shram S.I. (2020). Genetically Encoded Fluorescent Sensor for Poly-ADP-Ribose. Int. J. Mol. Sci..

[29] Guillemette S., Serra R.W., Peng M., Hayes J.A., Konstantinopoulos P.A., Green M.R., Cantor S.B. (2015). Resistance to therapy in BRCA2 mutant cells due to loss of the nucleosome remodeling factor CHD4. Genes Dev..

[30] Fouquerel E., Goellner E.M., Yu Z., Gagne J.P., Barbi de Moura M., Feinstein T., Wheeler D., Redpath P., Li J., Romero G. (2014). ARTD1/PARP1 negatively regulates glycolysis by inhibiting hexokinase 1 independent of NAD+ depletion. Cell Rep..

[31] Fang Q., Inanc B., Schamus S., Wang X.H., Wei L., Brown A.R., Svilar D., Sugrue K.F., Goellner E.M., Zeng X. (2014). HSP90 regulates DNA repair via the interaction between XRCC1 and DNA polymerase beta. Nat. Commun..

[32] Slyskova J., Sabatella M., Ribeiro-Silva C., Stok C., Theil A.F., Vermeulen W., Lans H. (2018). Base and nucleotide excision repair facilitate resolution of platinum drugs-induced transcription blockage. Nucleic Acids Res..

[33] Fang Q., Andrews J., Sharma N., Wilk A., Clark J., Slyskova J., Koczor C.A., Lans H., Prakash A., Sobol R.W. (2019). Stability and sub-cellular localization of DNA polymerase beta is regulated by interactions with NQO1 and XRCC1 in response to oxidative stress. Nucleic Acids Res..

[34] Sanjana N.E., Shalem O., Zhang F. (2014). Improved vectors and genome-wide libraries for CRISPR screening. Nat. Methods.

[35] Sykora P., Witt K.L., Revanna P., Smith-Roe S.L., Dismukes J., Lloyd D.G., Engelward B.P., Sobol R.W. (2018). Next generation high throughput DNA damage detection platform for genotoxic compound screening. Sci. Rep..

[36] Wang Z., Michaud G.A., Cheng Z., Zhang Y., Hinds T.R., Fan E., Cong F., Xu W. (2012). Recognition of the iso-ADP-ribose moiety in poly(ADP-ribose) by WWE domains suggests a general mechanism for poly(ADP-ribosyl)ation-dependent ubiquitination. Genes Dev..

[37] Donawho C.K., Luo Y., Luo Y., Penning T.D., Bauch J.L., Bouska J.J., Bontcheva-Diaz V.D., Cox B.F., DeWeese T.L., Dillehay L.E. (2007). ABT-888, an orally active poly(ADP-ribose) polymerase inhibitor that potentiates DNA-damaging agents in preclinical tumor models. Clin. Cancer Res..

[38] James D.I., Smith K.M., Jordan A.M., Fairweather E.E., Griffiths L.A., Hamilton N.S., Hitchin J.R., Hutton C.P., Jones S., Kelly P. (2016). First-in-Class Chemical Probes against Poly(ADP-ribose) Glycohydrolase (PARG) Inhibit DNA Repair with Differential Pharmacology to Olaparib. ACS Chem. Biol..

[39] Svilar D., Goellner E.M., Almeida K.H., Sobol R.W. (2011). Base excision repair and lesion-dependent subpathways for repair of oxidative DNA damage. Antioxid. Redox Signal..

[40] Royant A., Noirclerc-Savoye M. (2011). Stabilizing role of glutamic acid 222 in the structure of Enhanced Green Fluorescent Protein. J. Struct. Biol..

[41] Donnelly M.L.L., Hughes L.E., Luke G., Mendoza H., Ten Dam E., Gani D., Ryan M.D. (2001). The ‘cleavage’ activities of foot-and-mouth disease virus 2A site-directed mutants and naturally occurring ‘2A-like’ sequences. J. Gen. Virol..

[42] Rouleau M., Patel A., Hendzel M.J., Kaufmann S.H., Poirier G.G. (2010). PARP inhibition: PARP1 and beyond. Nat. Rev. Cancer.

[43] Buntz A., Wallrodt S., Gwosch E., Schmalz M., Beneke S., Ferrando-May E., Marx A., Zumbusch A. (2016). Real-Time Cellular Imaging of Protein Poly(ADP-ribos)ylation. Angew. Chem. Int. Ed..

[44] Wallrodt S., Buntz A., Wang Y., Zumbusch A., Marx A. (2016). Bioorthogonally Functionalized NAD(+) Analogues for In-Cell Visualization of Poly(ADP-Ribose) Formation. Angew. Chem. Int. Ed..

[45] Smith R., Timinszky G. (2018). Monitoring Poly(ADP-Ribosyl)ation in Response to DNA Damage in Live Cells Using Fluorescently Tagged Macrodomains. Methods Mol. Biol..

[46] Walker S., Landovitz R., Ding W.D., Ellestad G.A., Kahne D. (1992). Cleavage behavior of calicheamicin gamma 1 and calicheamicin T. Proc. Natl. Acad. Sci. USA.

[47] Li J., Saville K.M., Ibrahim M., Zeng X., McClellan S., Angajala A., Beiser A., Andrews J.F., Sun M., Koczor C.A. (2021). NAD(+) bioavailability mediates PARG inhibition-induced replication arrest, intra S-phase checkpoint and apoptosis in glioma stem cells. NAR Cancer.

[48] Gravells P., Grant E., Smith K.M., James D.I., Bryant H.E. (2017). Specific killing of DNA damage-response deficient cells with inhibitors of poly(ADP-ribose) glycohydrolase. DNA Repair.

[49] Houl J.H., Ye Z., Brosey C.A., Balapiti-Modarage L.P.F., Namjoshi S., Bacolla A., Laverty D., Walker B.L., Pourfarjam Y., Warden L.S. (2019). Selective small molecule PARG inhibitor causes replication fork stalling and cancer cell death. Nat. Commun..

[50] Schindelin J., Arganda-Carreras I., Frise E., Kaynig V., Longair M., Pietzsch T., Preibisch S., Rueden C., Saalfeld S., Schmid B. (2012). Fiji: An open-source platform for biological-image analysis. Nat. Methods.

