# Supplementary material for: Live Cell Detection of Poly(ADP-Ribose) for Use in Genetic and Genotoxic Compound Screens
Source: Cancers (Basel). 2022 Jul 28;14(15):3676. doi: 10.3390/cancers14153676 (PMC9367489; doi:10.3390/cancers14153676)
Supplement: Supplementary file 1 [file cancers-14-03676-s001.zip › cancers-1799612-supplementary.pdf]

Supplement information for:

Live Cell Detection of Poly(ADP)-Ribose for Use in Genetic and Genotoxic Compound Screens

Christopher A. Koczor [1,2], Aaron J. Haider [2], Kate M. Saville [1,2], Jianfeng Li [1,2], Joel F. Andrews [2], Alison V. Beiser [1,2], and Robert W. Sobol [1,2,*]

[1] Department of Pharmacology, College of Medicine, University of South Alabama, Mobile, AL 36688, USA; cakoczor@southalabama.edu (C.A.K.); aaronhai@uab.edu (A.J.H.); mkm1325@jagmail.southalabama.edu (K.M.S.); jianfengli@southalabama.edu (J.L.); jandrews@southalabama.edu (J.F.A.); avbeiser@southalabama.edu (A.V.B.)

[2] Mitchell Cancer Institute, University of South Alabama, Mobile, AL 36604, USA

* Correspondence: rwsobol@southalabama.edu (R.W.S.)

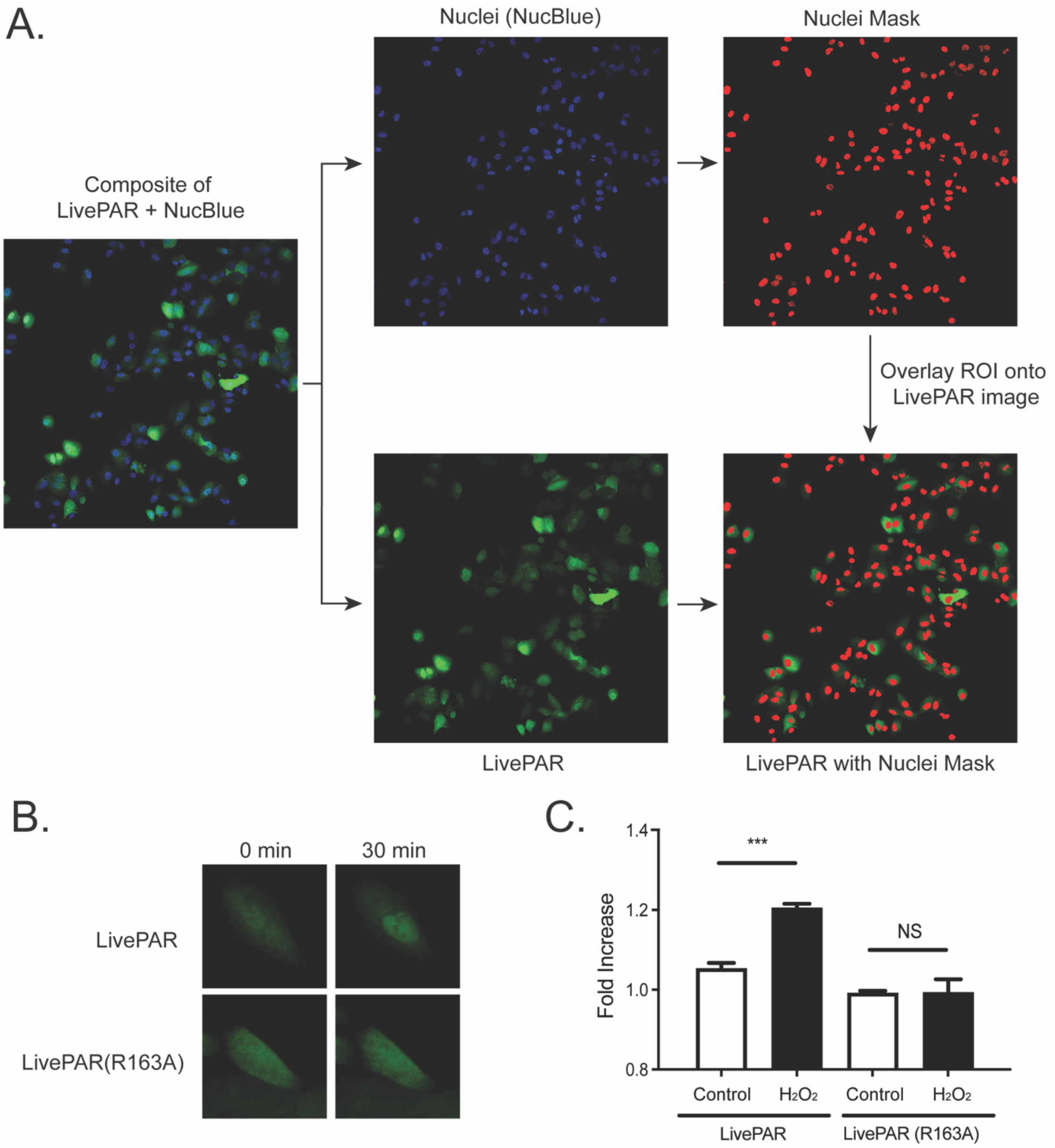

Figure S1. LivePAR nuclear enrichment assay. (**A**) Quantitative imaging approach for the LivePAR nuclear enrichment assay. A549 cells expressing LivePAR (green) were pre-treated with NucBlue live cell dye (blue). Images were taken at specific time points both before and after genotoxic treatment. Images from the blue channel were used to identify nuclei (ROIs) that were then used to quantify LivePAR nuclear intensity in the green channel. Each ROI was quantitated individually and were tracked for the duration of the experiment. (**B**) Images of LivePAR enrichment following 30 min H_2O_2 treatment (250µM) as compared to LivePAR(R163A). (**C**) Quantitation of LivePAR nuclear enrichment following 30 min H_2O_2 treatment (250µM). Nuclear enrichment was not observed in the LivePAR(R163A) mutant.

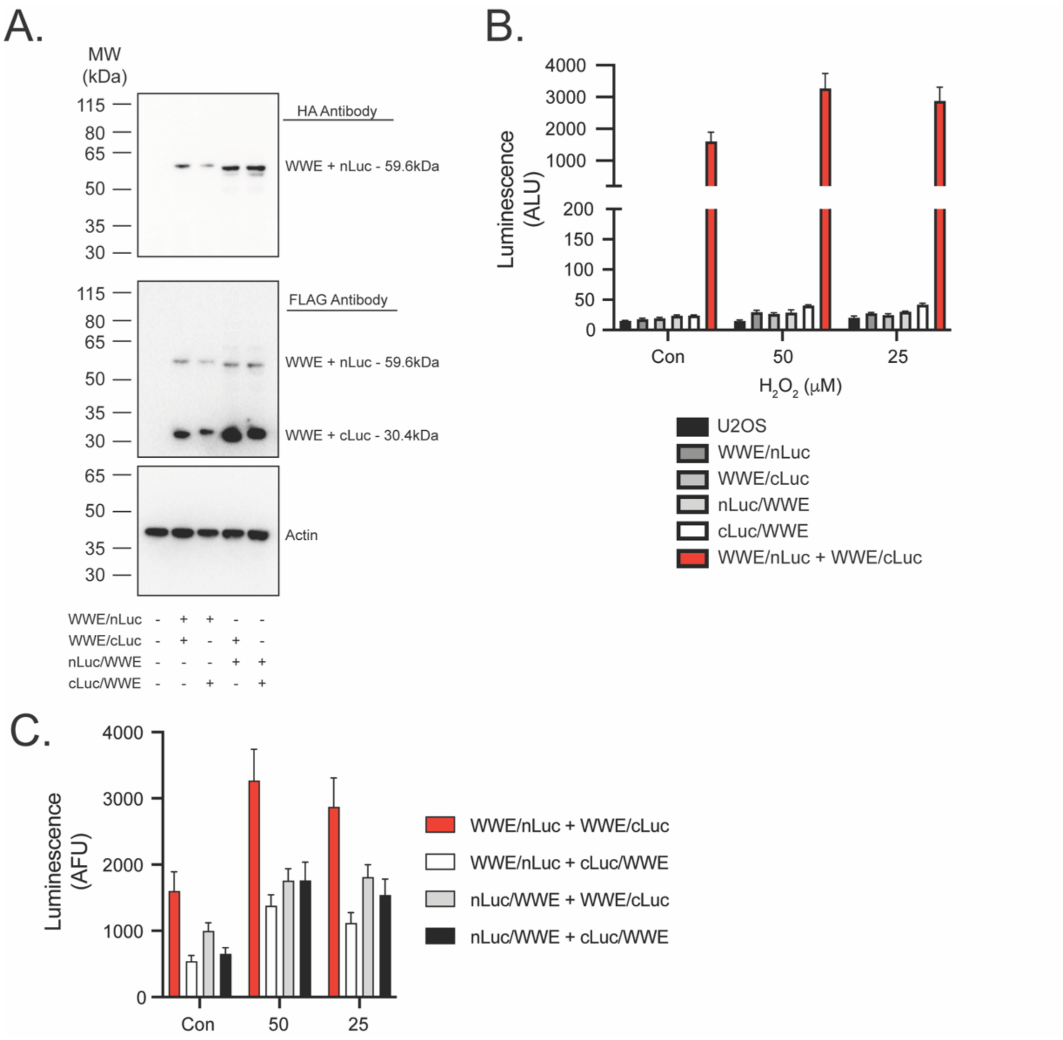

Figure S2. Comparison of WWE split luciferase (Luc) individual fusion pairings. (**A**) Expression of WWE fusions with either nLuc or cLuc in U2OS cells. WWE was fused to either the N-terminal side of nLuc or cLuc, or to the C-terminal side of nLuc or cLuc and cloned into single expression cassettes. Four combinations were tested in U2OS cells. (**B**) Raw luminescence of individual WWE split luciferase components tested. (**C**) Raw luminescence of WWE split luciferase pairs. WWE/nLuc paired with WWE/cLuc yielded the greatest total luminescence.

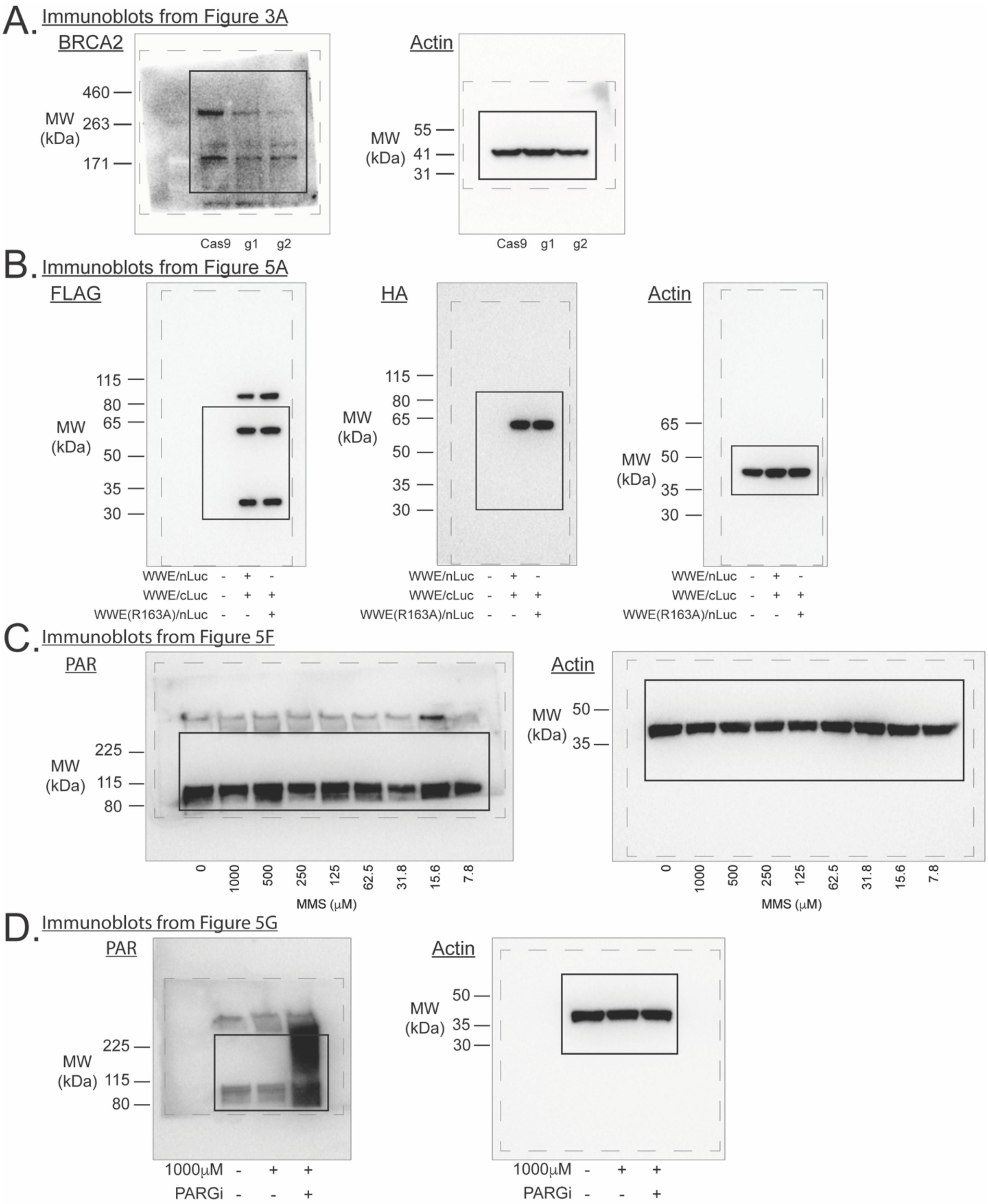

Figure S3. Uncropped immunoblot images. Each panel shows the uncropped images of the gel lanes used in the figure mentioned. Gray dotted lines denote the edge of the membrane analyzed; black lines denote the borders of the cropped image displayed in the main figure. (**A**) Raw data of immunoblots from Figure 3A. (**B**) Raw data of immunoblots from Figure 5A. (**C**) Raw data of immunoblots from Figure 5F. (**D**) Raw data of immunoblots from Figure 5G.

Table S1: Reagent List	SOURCE	IDENTIFIER
**Antibodies**		
Mouse anti-PAR (10H) (Immunoblot- 1:1000)	Generous gift from Mathias Ziegler (University of Bergen, Norway)	N/A
Mouse anti-beta actin (Immunoblot- 1:2500)	Sigma	Cat# A5441
Rabbit anti-BRCA2 (Immunoblot- 1:500)	Abcam	Cat# ab27976
Mouse anti-HA (Immunoblot- 1:2500)	Sigma	Cat# H3663
Mouse anti-FLAG M2 (Immunoblot- 1:2500)	Sigma	Cat# F1804
Immun-Star Goat anti-mouse-HRP conjugate (Immunoblot- 1:2500)	Bio-Rad	Cat# 170-5047
Immun-Star Goat anti-rabbit-HRP conjugate (Immunoblot- 1:2500)	Bio-Rad	Cat# 170-5046
**Chemicals, peptides, and recombinant proteins**		
Heat-inactivated fetal bovine serum	Bio-Techne	Cat# S11150H
Penicillin/streptomycin	Thermo Fisher Scientific	Cat# 15140-122
DMEM	Corning	Cat# 15-017-CV
L-glutamine	Thermo Fisher Scientific	Cat# 25030-081
Dimethyl Sulfoxide	Thermo Fisher Scientific	Cat# BP231-1
Puromycin	Sigma-Aldrich	Cat# P9620-10ml
Hygromycin	Thermo Fisher Scientific	Cat# 10687010
Trypsin-EDTA	Thermo Fisher Scientific	Cat# 25200-056
0.2μM PVDF	Bio-Rad	Cat# 162-0174
0.45μM Durapore Steriflip Filters	Sigma-Aldrich	Cat# SE1M003M00
Polybrene	Sigma-Aldrich	Cat# 107689
Protease inhibitor cocktail tablets	Thermo Fisher Scientific	Cat# 88666
Blotting grade non-fat dry milk	Bio-Rad	Cat# 170-6404
Nupage 4-12% Bis-Tris gel	Invitrogen	Cat# NP0323BOX
Nupage 3-8% Tris-Acetate gel	Invitrogen	Cat# EA0378BOX
Clarity Western ECL Substrate	Bio-Rad	Cat# 1705060
SuperSignal West Femto Maximum Sensitivity Substrate	Thermo Fisher Scientific	Cat# 34095
DC protein assay kit	Bio-Rad	Cat# 5000112
HT PARP In Vivo Pharmacodynamic ELISA Kit II	Bio-Techne	Cat# 4520B-096-K
Hydrogen Peroxide (9.8M)	Sigma-Aldrich	Cat# H1009
Methylmethanesulfonate (MMS)	Sigma-Aldrich	Cat# 129925
Formaldehyde solution (37%)	Thermo Fisher Scientific	Cat# BP531-500
NucBlue Live Cell ReadyProbes (Hoechst 33342)	Thermo Fisher Scientific	Cat# R37605
NucBlue Fixed Cell ReadyProbes (DAPI)	Thermo Fisher Scientific	Cat# R37606
Alexa Fluor 647 Phalloidin	Thermo Fisher Scientific	Cat# A22287
TransIT-X2 Transfection Reagent	Mirus Bio	Cat# MIR 6005
IVISbrite D-luciferin	Perkin Elmer	Cat# 122799
ABT-888 (Veliparib)	Selleckchem	Cat# S1004
PDD00017273	Sigma-Aldrich	Cat# SML1781
PDD00017238	Bio-Techne/Tocris	Cat# 7007
Bleomycin	Selleckchem	Cat# S1214

Hydroxyurea	Sigma	Cat# H8627
Calicheamicin	Thermo Fisher Scientific	Cat# 50-202-9283
AZD6738	Selleckchem	Cat# S7693
Doxorubicin	Selleckchem	Cat# S1208
Etoposide	Selleckchem	Cat# S1225
Gemcitabine	Selleckchem	Cat# S1714
MNNG	Sigma-Aldrich	Cat# 129941
**Cell Lines**		
U2OS<br>(Human osteosarcoma tumor cell line)	ATCC	Cat# HTB-96
A549<br>(Human adenocarcinoma tumor cell line)	ATCC	Cat# CCL-185
PEO1	Generous gift from Toshi Taniguchi (Fred Hutchinson CRC, USA)	[1]
C4-2	Generous gift from Toshi Taniguchi (Fred Hutchinson CRC, USA)	[1]
**Recombinant DNA**		
pLentiCRISPRv2<br>(Cas9 plus cloning site for gRNA; contains a puromycin resistance cassette)	[2]	Addgene (#52961)
pLentiCRISPRv2-Con<br>(Cas9 plus control gRNA; contains a puromycin resistance cassette)	Generous gift from Wim Vermeulen (Erasmus MC, NL)	[3]
pLENTI-CRISPR-V2-BRCA2-KO-g1<br>(Cas9 plus BRCA2 gRNA #1; contains a puromycin resistance cassette)	Generous gift from Wim Vermeulen (Erasmus MC, NL)	[3]
pLENTI-CRISPR-V2- BRCA2-KO-g2<br>(Cas9 plus BRCA2 gRNA #2; contains a puromycin resistance cassette)	Generous gift from Wim Vermeulen (Erasmus MC, NL)	[3]
pLV-EF1A-LivePAR-Hygro<br>(PAR binding domain with EGFP tag & a hygromycin resistance cassette)	[4]	Addgene (#176063)
pLV-EF1A-LivePAR(R163A)-Hygro<br>(EGFP fused to the C-terminus of a WWE domain containing a point mutation to convert Arg163 to Ala & a hygromycin resistance cassette)	This study	Addgene (#187609)
pLV-EF1A-WWE-HA-nLuc-FLAG-T2A-WWE-myc-cLuc-FLAG-IRES-Puro<br>(Split luciferase single construct with WWE-nLuc co-expressed with WWE-cLuc, with a puromycin resistance cassette)	This study	Addgene (#187611)
pLV-EF1A-WWE(R163A)-HA-nLuc-FLAG-T2A-WWE-myc-cLuc-FLAG-IRES-Puro<br>(Split luciferase single construct with WWE(R163A)-nLuc co-expressed with WWE-cLuc, with a puromycin resistance cassette)	This study	Addgene (#187612)

Software and Algorithms		
Image J	Image J 1.48v	https://imagej.nih.gov/ij/ 1.6.0_65
FIJI	http://fiji.sc/	[5]
Adobe Illustrator (for preparation of figures)	Adobe Systems	Version 2022
GraphPad Prism	GraphPad	Version 9 (Mac OS X)
MIDAS	[4]	https://doi.org/10.5281/zenodo.5534950
NIS-Elements	Nikon Instruments	Versions 4.51 and 5.11
Comet Analysis Software (CAS)	BioTechne	Version 1.3d

References

1. Sakai, W.; Swisher, E.M.; Jacquemont, C.; Chandramohan, K.V.; Couch, F.J.; Langdon, S.P.; Wurz, K.; Higgins, J.; Villegas, E.; Taniguchi, T. Functional restoration of BRCA2 protein by secondary BRCA2 mutations in BRCA2-mutated ovarian carcinoma. *Cancer Res* **2009**, *69*, 6381-6386, doi:10.1158/0008-5472.CAN-09-1178.
2. Sanjana, N.E.; Shalem, O.; Zhang, F. Improved vectors and genome-wide libraries for CRISPR screening. *Nat Methods* **2014**, *11*, 783-784, doi:10.1038/nmeth.3047.
3. Slyskova, J.; Sabatella, M.; Ribeiro-Silva, C.; Stok, C.; Theil, A.F.; Vermeulen, W.; Lans, H. Base and nucleotide excision repair facilitate resolution of platinum drugs-induced transcription blockage. *Nucleic Acids Res* **2018**, *46*, 9537-9549, doi:10.1093/nar/gky764.
4. Koczor, C.A.; Saville, K.M.; Andrews, J.F.; Clark, J.; Fang, Q.; Li, J.; Al-Rahahleh, R.Q.; Ibrahim, M.; McClellan, S.; Makarov, M.V.; et al. Temporal dynamics of base excision/single-strand break repair protein complex assembly/disassembly are modulated by the PARP/NAD(+)/SIRT6 axis. *Cell reports* **2021**, *37*, 109917, doi:10.1016/j.celrep.2021.109917.
5. Schindelin, J.; Arganda-Carreras, I.; Frise, E.; Kaynig, V.; Longair, M.; Pietzsch, T.; Preibisch, S.; Rueden, C.; Saalfeld, S.; Schmid, B.; et al. Fiji: an open-source platform for biological-image analysis. *Nat Methods* **2012**, *9*, 676-682, doi:10.1038/nmeth.2019.
